# Tyrosine Kinase Signaling in Kidney Glomerular Podocytes

**DOI:** 10.1155/2011/317852

**Published:** 2011-05-30

**Authors:** Seisuke Hattori, Shoichiro Kanda, Yutaka Harita

**Affiliations:** ^1^Division of Biochemistry, School of Pharmaceutical Science, Kitasato University, 5-9-1 Shirokane, Minato-ku, Tokyo 108-8641, Japan; ^2^Department of Pediatrics, Graduate School of Medicine, The University of Tokyo, 7-3-1 Hongo, Bunkyo-ku, Tokyo 113-8655, Japan; ^3^Department of Molecular Biology, Yokohama City University School of Medicine, Kanazawa-ku, Yokohama 236-0004, Japan; ^4^Division of Functional Proteomics, Graduate School of Nanobioscience, Yokohama City University Tsurumi-ku, Yokohama, Kanagawa 230-0045, Japan

## Abstract

During the last decade, several key molecules have been identified as essential components for the filtration barrier function of kidney glomerular podocytes. Mutations in genes encoding these molecules severely impair the podocyte architecture in the affected patients, leading to the development of proteinuria. Extensive investigations have been performed on the function of these molecules, which highlights the importance of tyrosine kinase signaling in the podocytes. An Src family tyrosine kinase, Fyn, plays a major role in this signaling pathway. Here, we review the current understanding of this important signal transduction system and its role in the development and the maintenance of podocytes.

## 1. Introduction

**Figure 1:**
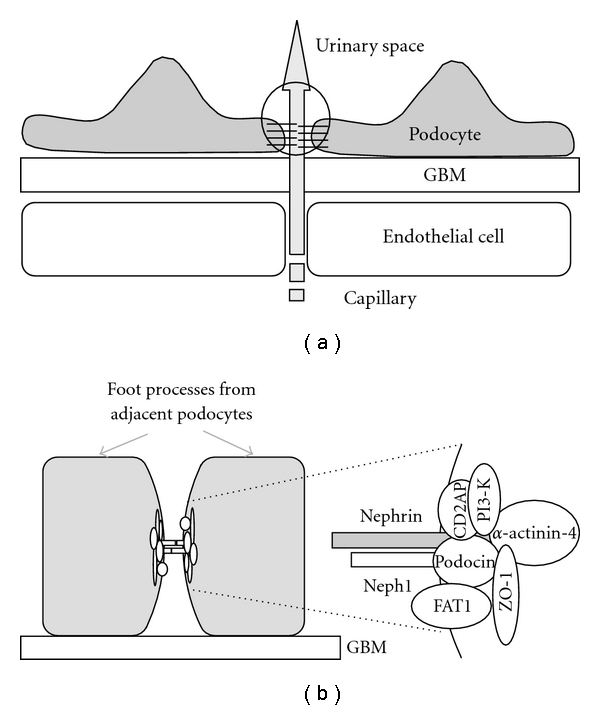
Schematic drawing of slit diaphragm (SD) of kidney glomerular podocytes. (a) The capillaries of kidney glomeruli are surrounded by three layers: the endothelial cells, the glomerular basement membrane (GBM), and the highly branched glomerular visceral epithelial cells, called podocytes. The blood pressure drives the filtration of blood plasma into the urinary space. Neighboring podocytes form a unique cell adhesion structure called SD that functions as a filtration barrier (enclosed by a circle). (b) Molecular structure of SD. The components of SD shown in this schematic drawing of SD have been identified by human and mouse genetics.

One of the major roles of kidney glomerulus is to filtrate blood plasma to excrete noxious metabolites into urine. A pair of kidneys filtrates around two hundred liters of blood a day, which is about fifty times whole blood volume. The glomerulus, the site of filtration, consists of a cluster of capillaries, which is surrounded by three layers; the endothelial cells, the glomerular basement membrane (GBM), and the highly branched glomerular visceral epithelial cells, called podocytes [1–4] (Figure 1(a)). A number of podocyte proteins are shown to be essential for glomerular filtration barrier function, indicating that podocytes play a major role in the filtration process. 

 Podocytes form primary protrusions that further extend numerous foot processes. Foot processes from neighboring podocytes interdigitate with each other and surround the entire surface of the capillary loops. The molecular architecture of these foot processes consists of highly organized actin filaments and actin filament-binding proteins [5]. These foot processes are bridged by a unique cell adhesion structure, the slit diaphragm (SD). It is SD that functions as a filtration barrier of glomerulus, allowing the passage of water and solutes of low molecular weight from the capillary lumen to the urinary space to form primitive urine, while restricting the flux of macromolecules. Useful materials including water, ions, and other nutrients are reabsorbed from the primitive urine through tubules into peritubular capillaries. 

 Components of SD have been identified by human and mouse disease genetics (Figure 1(b)). Nephrin, a central molecule of SD, was first identified as a product of *NPHS1* gene. The mutations in *NPHS1* gene cause early onset of heavy proteinuria and rapid progression to end-stage renal disease (congenital nephrotic syndrome of the Finnish type) [6, 7]. Nephrin is a transmembrane protein that belongs to the immunoglobulin superfamily. Neph1 is structurally related to Nephrin, and mice deficient in Neph1 develop proteinuria and die early after birth [8]. *NPHS2*, encoding the glomerular protein podocin, is mutated in the autosomal recessive steroid-resistant nephrotic syndrome [9]. Mutations in *ACTN4*, coding for *α*-actinin-4, cause familial focal segmental glomerulosclerosis (FSGS) [10]. Several other molecules, including FAT1 and CD2AP, are also identified as components of SD. The mutations in genes encoding these molecules in human diseases or in genetically manipulated mice result in similar phenotypic conditions: the flattening of foot processes (called effacement) and the loss of SD structure, leading to proteinuria ([7, 8, 11–13] reviewed in references [1–4]). 

 Nephrin interacts with Neph1 and podocin, forming a trimeric protein complex [14–17] (Figure 1(b)). These transmembrane proteins at SD further interact with the junctional scaffolding proteins, ZO-1 [18], CASK [19], and MAGI-1/2 [20, 21], *α*-actinin-4 [21] that anchor the SD complex to the elaborate actin cytoskeletal networks. Most of these proteins are crucial to both the development of the glomerulus and the filter function of SD. 

 In addition to its role as a structural framework of the filtration barrier, SD also plays an important role as a signaling platform [3, 4, 22]. Fin_minor_ patients, whose Nephrin cytoplasmic domain is deleted, exhibit a disease phenotype very similar to Nephrin null mutation [6], suggesting that Nephrin functions as a key molecule in this signaling complex. Indeed, Nephrin interacts with IQGAP [23], an effector protein of small GTPases Rac1 and Cdc42. Nephrin and CD2AP also interact with phosphatidylinositol 3-kinase (PI3-kinase) p85 subunit, which leads to an increased Akt activity and a reduction in cell death induced by apoptotic stimuli [24]. There are also lines of evidence that tyrosine phosphorylation may play a key role in the integrity of SD as described below.

## 2. Tyrosine Phosphorylation of Nephrin by Fyn

A significant portion of Nephrin resides in detergent resistant membrane fraction (DRM) [25, 26]. An antibody against podocyte-specific 9*-*O-acetylated GD3 ganglioside can immunoisolate Nephrin in DRM. Interestingly, *in vivo* administration of this antibody could induce effacement of SD, a hallmark of SD impairment, with a concomitant tyrosine phosphorylation of Nephrin [25]. In cultured cells, cross-linking of Nephrin with primary and secondary antibodies also induces Nephrin tyrosine phosphorylation [27]. Several nonreceptor tyrosine kinases including Src, Yes, Lyn, and Fyn are expressed in a podocyte-derived cell line, NPH15 [27]. These tyrosine kinases can phosphorylate Nephrin, when coexpressed with Nephrin in cultured cells [27, 28]. 

 The disruption of *fyn* gene causes proteinuria [29]. Using genetically engineered mice, Verma et al. clearly showed that Fyn is responsible for the phosphorylation of Nephrin in podocytes [26]. *In vitro* phosphorylation of Nephrin in the DRM fraction isolated from glomeruli of *fyn−/−* mice showed a greatly decreased tyrosine phosphorylation of Nephrin compared to that from wild-type mice. Fyn also binds to Nephrin in glomeruli. Furthermore, the structure of SD is severely impaired in *fyn*−/− mice [26]. Simultaneous disruption of another tyrosine kinase gene, *yes*, drives the phenotype severer, suggesting that Yes has an auxiliary role. These observations show that Fyn is a primary kinase for the tyrosine phosphorylation of Nephrin* in vivo* [26].

## 3. Nck Links Nephrin to Actin Polymerization

**Figure 2:**
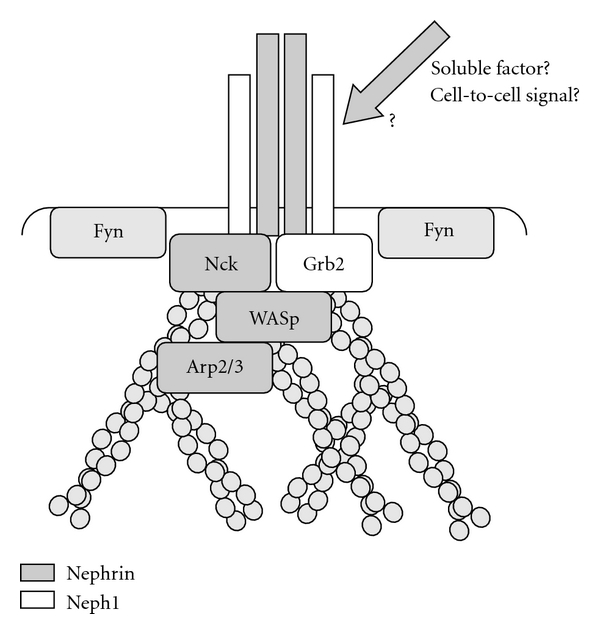
Nephrin and Neph1 cooperatively trigger actin polymerization. Upon tyrosine phosphorylation by Fyn, Nephrin and Neph1 recruit Nck and Grb2, respectively, that jointly initiate actin polymerization. For details, see text.

In 2006, two groups independently reported that phosphorylation of Nephrin cytoplasmic domain recruits an SH2 (Src homology 2)-SH3 containing adaptor protein, Nck [30, 31]. Nck binds to phosphorylated tyrosine residues, Y1191, Y1208, and Y1232 of Nephrin (residues are numbered based on the mouse Nephrin sequence, accession number AAK38483). Amino acid sequences surrounding all of these three tyrosine residues fulfill the preferential binding motif for Nck SH2 (Tyr-Asp-X-Val) [32], and these residues are phosphorylated by Fyn *in vitro* [30, 33]. It is well documented that Nck plays an essential role in the initiation of actin polymerization. Nck has one SH2 and three SH3 domains. By recruiting more than 20 SH3 binding proteins to activated receptors or substrates for the activated receptors, Nck regulates dynamic actin cytoskeletal organization and motogenic responses (reviewed in reference [34]) (Figure 2). For example, IRS-1 and Sos are involved in the stimulation of cell growth and WASp and Pak are major regulators of actin cytoskeleton. 

 Nck has closely related two members, Nck1 and Nck2, both of which are expressed in podocytes. Consistently, mice lacking either *Nck1* or *Nck2* are viable without apparent renal defects, and those lacking both genes are embryonic lethal at day 9.5 [31]. Therefore, Jones et al. selectively disrupted *Nck2* in podocytes by means of *Cre-loxP* system in *Nck1−/−* background [31]. These mice developed proteinuria and focal sclerosis in glomeruli after birth. Electron microscopic examination of four-day-old pups' kidney revealed complete fusion of foot processes, that is, the loss of SD structure. Clustering of Nephrin expressed in mouse embryonic fibroblasts from *Nck1*−/−*Nck2*−/− mice induced localized actin polymerization in a coexpressed Nck2-dependent manner [31]. Nck function is still necessary even in adult mice, because inducible deletion of Nck in podocytes results in proteinuria, glomerulosclerosis, and altered morphology of foot processes [42].

 WASp/Scar proteins are important regulators of actin cytoskeleton that promote actin filament nucleation and branching by direct binding of their C termini to the actin-related protein 2 and 3 (Arp2/3) complex [43]. The interaction of WASp/Scar proteins with the Arp2/3 complex is regulated by a number of activators, including the Rho-family GTPases, phosphoinositides, and SH3 domains [44]. Nck SH3 domains can induce, in cooperation with phosphatidylinositol 4,5-bisphosphate (PIP2), full activation of actin nucleation in an *in vitro* reconstituted system containing N-WASp-Arp2/3 complexes in the absence of Cdc42 [45]. An essential role of Nck in mediating pathogen-induced actin filament assembly *in vivo* is also shown [46].

 The role of ligands of tyrosine kinase receptors is to dimerize the receptors to activate their tyrosine kinase activity [47]. Consistent with this notion, it is known that antibodies against the extracellular domain of tyrosine kinase receptors can mimic the role of their native ligands. As to Nephrin, however, the application of the primary antibody against Nephrin alone is not sufficient for Nephrin to become tyrosine phosphorylated [27]. In addition, the secondary antibody against the primary antibody is necessary, indicating that clustering/aggregation of Nephrin is required to be phosphorylated by Fyn. Under such conditions, the local concentration of Nck recruited to clustering Nephrin may become high enough to induce actin polymerization. In this respect, it is worth mentioning that a chimeric protein consisting of extracellular and transmembrane domain from CD16 and CD7, respectively, fused to Nck is sufficient to induce actin polymerization upon clustering [48]. However, the timing and the location of Nephrin phosphorylation, Nck recruitment, and following actin polymerization *in vivo* still remain unclear at present. Therefore, this possibility requires further examination in experimental animal models. Verma et al. reported that strong phospho-Nephrin signal is observed in developing glomeruli of mice, whereas it becomes weaker in adult tissues [30]. This observation suggests that Nephrin phosphorylation is required for the initiation of actin polymerization during junctional formation.

 Besides Nck-dependent regulation of actin polymerization, Nephrin may also regulate actin dynamics by other signaling pathways. Nephrin directly binds to IQGAP1 [23], an effector of Rac and Cdc42 that are involved in a wide range of cell biological processes, such as cell motility, polarity formation, and morphology [49]. Nephrin and CD2AP collaboratively control PI3-kinase-Akt pathway. In addition to suppressing proapoptotic signals [24], recent findings highlighted the role of PI3-kinase regulation of an actin filament-severing factor, cofilin1 [50, 51]. Nephrin activation induced an activation of cofilin1 (dephosphorylation of cofilin1), through the activation and inactivation of PI3-kinase and LIMK (LIM domain containing kinase), respectively. Podocyte-specific targeting of *Cfl1* in mice resulted in persistent proteinuria by 3 months of age [50]. Cofilin1 deficiency led to foot process effacement and proteinuria in zebrafish, and an accumulation of F-actin fibers and significantly decreased podocyte migration ability in cultured mouse podocytes [51]. Interestingly, unphosphorylated active cofilin1 was distributed throughout the cells in normal kidney tissues, whereas cofilin1 was inactivated by phosphorylation and observed in the nuclei of podocytes under glomerular disease conditions [51].

## 4. Collaboration of Nephrin and Neph1 in Actin Polymerization

Nephrin interacts with Neph1 by their cytoplasmic domains. In addition, Nephrin extracellular domain interacts with itself and Neph1 via a transinteraction between the adjacent foot processes [15, 16]. Neph1 is also a substrate for Fyn and binds Grb2 upon phosphorylation [40, 41]. Grb2 is another SH2-SH3 adaptor protein, recruiting SH3-binding partners to plasma membrane. Unlike Nephrin that interacts with Nck at three binding sites, Neph1 seems to provide only one binding site for Grb2 [40, 41]. 

 Garg et al. constructed a chimeric protein consisting of CD16 extracellular and CD7 transmembrane domains and Neph1 cytoplasmic domain (CD16/CD7/Neph1CD), and expressed the chimeric protein in NIH3T3 cells. Similar to Nephrin clustering, CD16/CD7/Neph1CD became tyrosine phosphorylated upon clustering and recruited Grb2, which triggered actin polymerization at the clustered sites. Neph1 phosphorylation required Fyn, since Neph1 phosphorylation did not occur in mouse embryonic fibroblasts obtained from *src*−/−*yes*−/−*fyn*−/− mice, which was rescued by transduction of *fyn* gene [40]. Because NIH3T3 cells do not express Nephrin, this clustering-induced phosphorylation of Neph1 seems to be Nephrin independent. Actin polymerization induced by Nephrin clustering was significantly augmented by co-expression of Neph1 suggesting their cooperative roles [40] (Figure 2). Since Nephrin and Neph1 interact each other, Nck and Grb2 may be positioned on the plasma membrane side by side at rather high concentrations. Interestingly, vaccinia virus has a similar actin polymerization mechanism. The viral protein A36R is tyrosine phosphorylated by Fyn that results in the recruitment of both Nck and Grb2 that cooperate to induce localized actin filament polymerization [52].

## 5. Tyrosine Kinase Signaling, Yet More

**Figure 3:**
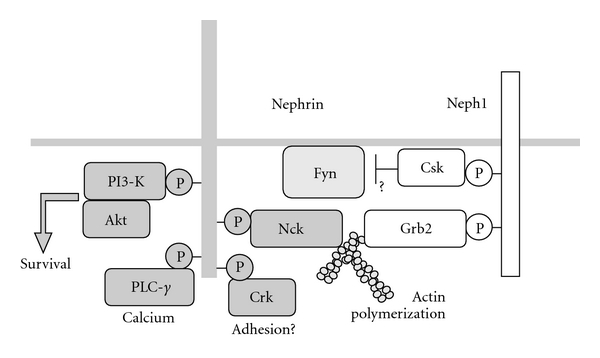
Signaling molecules in SD. In addition to Nck and Grb2 recruitment in Figure 2, Nephrin and Neph1 bind to signaling molecules shown in the figure to regulate a variety of cellular events.

Other SH2 containing molecules including Crk, ShcA, Fyn, p85 subunit of PI3-kinase, PLC-*γ*1, and Crk-L also bind to tyrosine phosphorylated but not unphosphorylated Nephrin [24, 30, 33] (Figure 3). PI3-kinase/Akt pathway also controls actin dynamics through a small GTPase Rac [53]. Stable transfection of rat Nephrin in the podocytes with podocin led to Nephrin tyrosine phosphorylation, PI3-kinase-dependent phosphorylation of Akt, increased Rac1 activity, and an altered actin cytoskeleton with decreased stress fibers and increased lamellipodia [53]. Crk also regulates cell adhesion to the basement membrane in collaboration with p130Cas by a C3G-Rap1 pathway in fibroblasts [54]. ShcA and PLC-*γ* are common players that mediate signals from various tyrosine kinase receptors. As in these systems, the binding of PLC-*γ* to phosphorylated Nephrin triggers calcium signaling pathways [33]. A phospho-peptide surrounding Nephrin Y1208 could precipitate PLC-*γ* 1 from the cell lysates. However, this result does not exclude the possibility that some factor mediates the association between Nephrin and PLC-*γ* 1. Mutations in PLC-*ε1* gene also cause early-onset nephrotic syndrome [55], suggesting an important role of calcium signaling in podocytes. 

 Tyrosine phosphorylation of Nephrin may also regulate the turnover of Nephrin. Nephrin is rapidly subjected to clathrin-mediated endocytosis or clathrin-independent, raft-mediated endocytosis [56]. Interaction of Nephrin with *β*-arrestin2 is diminished by phosphorylation of Y1208, which may result in the attenuation of clathrin-mediated endocytosis of Nephrin [57]. In contrast, Y1191F/Y1208F mutant of Nephrin lacking major phosphorylation sites is endocytosed at a slower rate than wild-type Nephrin in a raft-mediated manner, suggesting that phosphorylation of these sites augments the raft-mediated internalization [56]. Whether internalized Nephrin is still active in the signaling or whether recycling of Nephrin occurs or not awaits further experiments. 

 The recruitment of Grb2 may serve another function. Contrary to our expectation that the recruitment of Grb2 to the plasma membranes activates Ras/Erk pathway, Neph1 binding of Grb2 suppressed the activation of ERK [41]. Similar suppression of Erk activation by sprouty [58] or Dok-3 [59] is described. These molecules, when tyrosine phosphorylated, bind to Grb2 and may sequestrate Grb2/Sos complex into a compartment where Ras does not exist. It is, thus, possible that the Neph1 and Ras are localized in different membrane compartments, which needs to be confirmed by further experiments. Neph1 also binds CSK upon phosphorylation likely through its SH2 domain [41]. CSK is a member of Src family tyrosine kinase (SFK) that negatively regulates SFKs by phosphorylating their regulatory tyrosine residue [60]. Like Nephrin and Neph1, CSK is shown to reside in lipid raft [60]. The physiological meaning of this Neph1-CSK interaction is to be clarified.

## 6. Phosphorylation of SD Components during Injury of Podocytes

Proteomic analysis of glomeruli reveals that several SD components including Nephrin, Neph1, signal-inhibitory regulatory protein (SIRP)-*α* )SHPS-1), FAK1, and paxillin are tyrosine phosphorylated to some extent in normal glomeruli of adult rats [61]. But, how are SD components tyrosine phosphorylated? Or when their phosphorylation level is altered? This most important issue is still open to be investigated. Although clustering/aggregation of Nephrin induces Fyn-dependent phosphorylation in cultured cells, and this procedure may not reflect the pathogenesis of podocyte damage observed *in vivo*. Nephrin molecules interact with each other not only in *cis* (side by side) but also in *trans* (head-to-head) orientation [15]. Antibody cross-linking could reflect only the lateral assembly of Nephrin. Molecular basis for *in vivo* phosphorylation and dephosphorylation of Nephrin is thus still unclear; however, phosphorylation level of SD components has been investigated in various situations *in vivo*.

 Uchida et al. reported that phosphorylation of Nephrin in glomeruli of patients with minimal change nephrosis (MCN) is significantly decreased compared with that in normal glomeruli [35] (Table 1). The same study group also examined Nephrin phosphorylation in patients with membranous nephropathy (MN) [36]. A decrease in the immunofluorescent intensity of phospho-Nephrin is not observed in stage I, and only a slight decrease is seen in stages II, III, and IV compared with controls. However, no significant correlation between Nephrin phosphorylation and proteinuria is observed [36].

 Nephrin and Neph1 has been examined in several animal models that induce protein uria. However, inconsistent results are published so far (Table 1). Uchida et al. observed that Nephrin phosphorylation was visible before the puromycin aminonucleoside (PAN-) induced nephrosis, which became undetectable during the disease induction, with a concomitant decrease in filamentous actin contents [35]. Jones et al. [42] and Li et al. [37] also observed a similar decrease using the same animal model. On the other hand, Garg et al. [40] and Harita et al. [41] observed an increase in the phosphorylation of Nephrin and Neph1 in PAN-induced nephrosis.

 Recently, an adaptor protein, c-mip has received much attention [38]. c-mip, expression is invisible in healthy kidney whereas its expression is significantly upregulated in patients with MCN and MN. Interestingly, c-mip inhibits interactions between Fyn and the cytoskeletal regulator N-WASP (neural Wiskott-Aldrich syndrome protein) and between the adaptor protein Nck and Nephrin, which may account for the cytoskeletal disorganization and the effacement of foot processes, in mice or cultured podocytes overexpressing c-mip, phosphorylation of Nephrin decreases [38]. The same study also demonstrates that Nephrin phosphorylation also decreases in glomeruli injured by lipopolysaccharide treatment [38]. 

 In other experimental models such as protamine sulfate-nephritis or passive Heymann nephritis, opposite results are reported [25, 30, 33, 39, 41]. Perfusion of mouse kidneys with protamine sulfate (PS) results in foot process effacement within 15 min after the perfusion, while subsequent perfusion with heparin sulfate largely restores the normal podocyte morphology. Nephrin phosphorylation was observed in the podocytes of PS-perfused kidneys, which is rapidly reversed after heparin sulfate perfusion [30]. We observed similar upregulation in the tyrosine phosphorylation of Nephrin, Neph1, and PLC-*γ*1 in PS-perfused glomeruli ([33, 41], and Harita et al., unpublished results). The PS-induced phosphorylation sites are identical to those phosphorylated by Fyn [30, 33, 41]. Li et al. described the increase in both Nephrin phosphorylation and activity of Src family kinases in passive Heymann nephritis. These seemingly contradictory results may reflect differences in the experimental conditions. Progression of podocyte damage may differ among the animal models, therefore, the changes in the intracellular signaling responding to the damage may also differ. The specificity and sensitivity of antibodies used in these studies may also account for the difference. Commercial antiphosphotyrosine antibodies (pTyr) were used in some studies [37, 39, 40, 42], whereas homemade site-specific antibodies were used in other studies [30, 33, 35, 36, 38, 40–42]. Phosphorylation of Nephrin in human specimen is investigated in only a subset of nephrotic syndromes. Therefore, detailed analyses of various types of the disease, and the specific animal model that mimics each type of the disease, is necessary to clarify the phosphorylation status under the disease conditions.

## 7. Protein Tyrosine Phosphatases in Podocytes

*In vitro* treatment of podocytes with pervanadate, an inhibitor of protein tyrosine phosphatases, induces structural changes of actin cytoskeleton and focal contact similar to those induced by *in vivo* application of PS or PAN, suggesting that protein tyrosine phosphatases (PTPs) also regulate the podocyte process structure [37]. Podocytes express several protein tyrosine phosphatases, SHP-1/2, PTP-PEST, PTP-1B, PTP-36, and GLEPP1/PTPro [61–64]. GLEPP1/PTPro depletion in mice decreases the glomerular filtration rate with a change in podocyte morphology [63], and antibodies to GLEPP1/PTPro increase the permeability of albumin of isolated glomeruli *in vitro *[64], suggesting protein tyrosine phosphatases also modulate the podocyte filtration function. SIRP-*α*, a type I transmembrane glycoprotein, that recruits SHP-1/2 to the plasma membrane, is concentrated in SD of podocytes [65]. These results suggest that tyrosine phosphatases also play a significant role to maintain the appropriate phosphorylation level of SD components. 

 Our preliminary results have shown that tyrosine phosphorylation of Nephrin, Neph1, and PLC-*γ*1 is inversely correlated with that of SIRP-*α* during rat PS-induced podocyte injury models ([33, 41], and Harita et al. unpublished results). After the perfusion of PS, tyrosine phosphorylation of these proteins was significantly induced whereas the intense phospho-SIRP-*α* signal observed prior to the PS treatment became invisible. One possible scheme is that SHP-1/2 may detach from SIRP-*α* upon dephosphorylation of SIRP-*α*, shifting the phosphorylation/dephosphorylation balance toward phosphorylation, which may induce the phosphorylation of these molecules.

## 8. Calcium Signaling Controlled by Tyrosine Phosphorylation

PLC-*γ*1 binds to tyrosine phosphorylated Nephrin. The binding site of PLC-*γ*1 is Y1208, which is identical to one of the Nck binding sites [33]. Upon binding to tyrosine phosphorylated Nephrin PLC-*γ*1 becomes tyrosine phosphorylated and activated, with a concomitant increase in the inositol-1,4,5-tris phosphate (IP3) and intracellular calcium concentration [Ca^2+^]i [33]. IP3 is known to mobilize calcium from the internal stores, which triggers calcium efflux from the extracellular space. PLC-*γ* may also regulate actin dynamics by its product, phosphatidylinositol 4,5-bisphosphate (PIP2) [66]. Mutations in *PLC-*ε1** gene cause renal failure, suggesting an essential role of phosphoinositide signaling pathways in podocytes [55]. PLCE1 binds to IQGAP1, which is a binding partner of Nephrin [21]. *PLC-*ε1** gene expression begins in the developing glomerulus at rather early stage (S-shaped stage) of glomerular development and highly expressed during the early capillary loop stage. Morpholino oligonucleotide-mediated knockdown of *PLC-*ε1** caused to nephrotic syndrome in zebrafish [55]. 

 Podocyte damage can be caused by a variety of Ca^2+^-stimulating vasoactive hormones, including angiotensin II, bradykinin, and endothelin, through altering the glomerular ultrafiltration coefficient by Ca^2+^- and cAMP-dependent signals [67]. Recent finding that mutations in *TRPC6* gene are associated with a human proteinuric kidney disease, focal segmental glomerulosclerosis (FSGS) [68, 69] also links calcium signaling to podocyte injury. In this disease, podocytes and the SD lose their integrity, disrupting the glomerular filtration barrier [2–4]. TRPC6 is one of the mammalian homologues of *Drosophila* transient receptor potential canonical, the TRPCs, which are potent plasma membrane channels that contribute to changes in the cytosolic-free Ca^2+^ concentration, either by acting as Ca^2+^ entry pathways on the plasma membrane or by modulating the membrane driving force for Ca^2+^ entry through changing the membrane potential [70–72]. These proteins form a nonselective cation channel that is activated by receptor stimulation or by the exogenous application of diacylglycerol analogs. Among the seven mammalian TRPC channels, a subfamily of TRPC3, 6, 7 can be defined by their similarity in the primary structure and function. 

 Whereas the mode of heredity of *NPHES1* (Nephrin) and *NPHES2* (podocin) muatiions is recessive, the disease causing mutations of *TRPC6* gene are inherited in an autosomal dominant manner, which suggests that the mutations may enhance the channel activity. Increased expression of wild-type TRPC6 is also a common feature of human proteinuric kidney diseases [73]. While overexpression of both wild and mutant TRPC6 in podocytes in mice induces proteinuria [74], how the disease-causing mutations affect the channel activity of TRPC6 remains unclear. Some mutations (P112Q, R895C, E897K) enhance angiotensin II receptor-mediated activation of TRPC6 when expressed in HEK293 cells, while neither the S270T nor the N143S missense mutations, nor a 57-amino acid truncation mutation (K874X), altered the channel activity [68, 69]. These channel activities correlated well with the extent of downstream NFAT activation, mediated by a calcium dependent phosphatase, calcineurin [75]. On the other hand, the P112Q mutation increased the plasma membrane expression of TRPC6 [68], suggesting changes in surface expression may also contribute to the pathogenesis of the disease. 

Our recent unpublished findings concerning the role of TRPC6 phosphorylation may shed lights on this apparently puzzling issue (Kanda et al. unpublished data). Fyn also phosphorylates TRPC6 and enhances its channel activity [76]. Upon phosphorylation by Fyn at Y31 and Y284, TRPC6 forms a complex with PLC-*γ*1. This complex formation is critical for TRPC6 to translocate from cytoplasmic vesicles to the surface of plasma membrane, because siRNA-mediated downregulation of RLC-*γ*1 abolished the TRPC6 surface expression. Concomitant with this translocation, TRPC6 channel activity was enhanced. We also found that Nephrin binds to phosphorylated TRPC6, interfering with TRPC6-PLC-*γ*1 complex formation and subsequent translocation to the plasma membrane. Y284 of TRPC6 is also critical for Nephrin-TRPC6 complex formation. TRPC6 expression did not affect on Nephrin phosphorylation (either wild-type or mutants). Importantly, all the disease-causing mutations dramatically reduce the affinity of TRPC6 to Nephrin, which renders mutant TRPC6s insensitive to Nephrin suppression. Therefore, the proportion of surface expressed mutant TRPC6s is much higher than that of the wild-type channel, which may cause exaggerated calcium signaling in podocytes.

## 9. Conclusion

Kidney glomerular podocytes form an elaborate cell-to-cell adhesion structure called the slit diaphragm (SD) between adjacent podocytes. SD not only functions as a filtration barrier, but also plays an important role as a signaling scaffold. Human and mouse genetics as well as biochemical and cell biological studies have identified a variety of SD components. The disruption of genes encoding these components cause proteinuria in affected human patients and mice. 

 Tyrosine phosphorylation is a fundamental reaction that controls the integrity and function of SD. A tyrosine kinase, Fyn, phosphorylates several SD components including Nephrin, Neph1, and TRPC6. Upon phosphorylation, Nephrin recruits an adaptor protein Nck, which initiates actin polymerization. Neph1 also collaborates with Nephrin by recruiting another adaptor protein Grb2. Nephrin and Neph1 also bind to a wide range of signaling molecules upon tyrosine phosphorylation such as PLC-*γ*1, ShcA, PI3-kinase, Crk, and CSK, which induces further signaling to downstream molecules. Protein tyrosine phosphatases may also participate in this tyrosine phosphorylation signaling system.

*In vivo* conditions where tyrosine phosphorylation occurs still remain to be investigated. The most important issue is then to elucidate the molecular mechanism for the regulation of Fyn activity; what kinds of signals do control Fyn? Cell-to-cell interaction, some soluble factors, signals from the basement membrane, or cell-intrinsic signals? Insights into these questions may provide us the basis for the development of therapeutic approaches in future. 

 Lines of evidence suggest that oversignaling of calcium is toxic to podocytes. Mutations affecting TRPC6 calcium channel are found in patients with FSGS, which further supports the involvement of calcium signaling in the podocyte damage. Our unpublished results have shown that the disease causing mutations of TRPC6 significantly reduce the affinity of TRPC6 with Nephrin and promote its surface expression and channel activity, which may result in the oversignaling of calcium.

## Figures and Tables

**Table 1:** Summary of the studies on Nephrin and Neph1 phosphorylation under disease conditions.

	Antibodies	Phosphorylation in animal models	Phosphorylation in humann diseases	Reference
Nephrin	1208 and 1232	Decrease in PAN	Decrease in MCNS	[35]
	1208 and 1232		Slight decrease in MN	[36]
	1232	Decrease in PAN		[35]
	pTyr	Decrease in PAN		[37]
	1191/1208	Decrease in LPS		[38]
	1191/1208	Decrease in c-mip*		[38]
	1191/1208	Increase in PAN		[30]
	1208	Increase in PS		[30]
	1208	Increase in PS		[33]
	pTyr	Increase in 27A**		[25]
	pTyr	Increase in PHN		[39]

Neph1	pTyr	Increase in PAN		[40]
	637/638	Increase in PAN, PS		[41]

The numbers in the column “antibodies” mean the site of phosphorylation based on the mouse Nephrin sequence (accession number AAK38483). “1208 and 1232” means separate antibodies and “1191/1208” antibody recognizes both phosphorylaion sites. pTyr means commercial antiphosphotyrosine antibodies. PAN: puromycin aminonucleoside; LPS: lipoporysaccharide; PS: protamine sulfate; PHN: passive Heymann nephritis. MCN: minimal change nephrosis; MN: membranous nephropathy. *Mice overexpressing c-mip; **mice injected with 27A antibody against podocyte-specific 9-O-acetylated GD3 ganglioside.
